# A Novel Three-Dimensional Vector Analysis of Axial Globe Position in Thyroid Eye Disease

**DOI:** 10.1155/2017/7253898

**Published:** 2017-04-09

**Authors:** Jie Guo, Jiang Qian, Yifei Yuan, Rui Zhang, Wenhu Huang

**Affiliations:** ^1^Department of Ophthalmology, Eye & ENT Hospital, Fudan University, Shanghai, China; ^2^Department of Radiology, Eye & ENT Hospital, Fudan University, Shanghai, China

## Abstract

*Purpose*. To define a three-dimensional (3D) vector method to describe the axial globe position in thyroid eye disease (TED). *Methods*. CT data from 59 patients with TED were collected and 3D images were reconstructed. A reference coordinate system was established, and the coordinates of the corneal apex and the eyeball center were calculated to obtain the globe vector $\vec{EC}$. The measurement reliability was evaluated. The parameters of $\vec{EC}$ were analyzed and compared with the results of two-dimensional (2D) CT measurement, Hertel exophthalmometry, and strabismus tests. *Results*. The reliability of $\vec{EC}$ measurement was excellent. The difference between $\vec{EC}$ and 2D CT measurement was significant (*p* = 0.003), and $\vec{EC}$ was more consistent with Hertel exophthalmometry than with 2D CT measurement (*p* < 0.001). There was no significant difference between $\vec{EC}$ and Hirschberg test, and a strong correlation was found between $\vec{EC}$ and synoptophore test. When one eye had a larger deviation angle than its fellow, its corneal apex shifted in the corresponding direction, but the shift of the eyeball center was not significant. The parameters of $\vec{EC}$ were almost perfectly consistent with the geometrical equation. *Conclusions*. The establishment of a 3D globe vector is feasible and reliable, and it could provide more information in the axial globe position.

## 1. Introduction

Globe position is a key element in the diagnosis and treatment of orbital diseases, especially thyroid eye disease (TED). Exophthalmos is the most widely used indicator in TED. Although different measurement methods have been proposed, all of them assess the axial position of the corneal apex and represent the change in the anterior-posterior direction only. Additionally, the computed tomography (CT) exophthalmos measurement, which is considered the reference standard, needs further evaluation for its reliability and accuracy [1–5]. Furthermore, the indicators to assess the axial globe position in the superior-inferior and medial-lateral directions are also important, but in TED, they are not uniform. Alsuhaibani et al., Takahashi and Kakizaki, and Fichter et al. have all reported that the globe position changes horizontally in TED patients, based on different measurement methods [6–8].

Strabismus is not uncommon in TED, meaning that the eyeballs may have a tilt change in these patients, in addition to protrusion or translation. On the one hand, there might be some deviation if we define the position of the whole eyeball by the corneal point only; on the other hand, it could not describe the orientation of the globe. As mentioned above, it might be better to evaluate the globe position in three-dimensional (3D) space using a vector, which could show the location as well as the orientation.

Describing the globe position with a 3D vector method could be also useful for orbital decompression surgery in TED. Having a more accurate measurement method could help evaluate postoperative changes of the axial globe position in different directions. What is more, new onset strabismus is an important complication after orbital decompression surgery, and the 3D vector method might help in studying the eyeball motion and tilt change.

The 3D reconstruction technique provides a more comprehensive view of the orbit and improves the ability to analyze in orbital diseases [9, 10]. In the following study, we established a coordinate system based on 3D CT images, defined the globe vector, and discussed the reliability of this method. We also compared the globe vector with the results of traditional two-dimensional (2D) CT measurement, Hertel exophthalmometry, and clinical strabismus tests.

## 2. Patients and Methods

### 2.1. Patients

Eighty-one sets of CT data from 59 patients diagnosed with TED were collected between 2012 and 2016 at the Eye and ENT Hospital of Fudan University. There were 30 females and 29 males, and the average age was 48.6 ± 11.3 years (ranging from 21 to 68 years). The exclusion criteria included the patients with orbital rim or zygomatic change from fracture or surgery, orbital tumor, and orbital inflammation. The research adhered to the tenets of the Declaration of Helsinki and was approved by the Ethics Committee of Fudan University. Informed consent was obtained from all the patients.

### 2.2. CT Scan and 2D Exophthalmos Measurement

CT scans were obtained at contiguous 0.75 mm thickness (SOMATOM Sensation, Siemens AG, Germany). The patients were instructed to look straight with fixed eyes during scanning. CT data were recorded in DICOM format (Digital Imaging and Communications in Medicine) and archived on compact discs, then imported to MIMICS 16.0 software (Materialise Dental, Leuven, Belgium) for image analysis.

The axial image including the thickest lens was chosen, and a line between the lateral orbital rims was drawn; then, the perpendicular distance between the corneal apex and this line was defined as the 2D CT exophthalmos.

### 2.3. Establishment of Globe Vector

**Figure 1:**
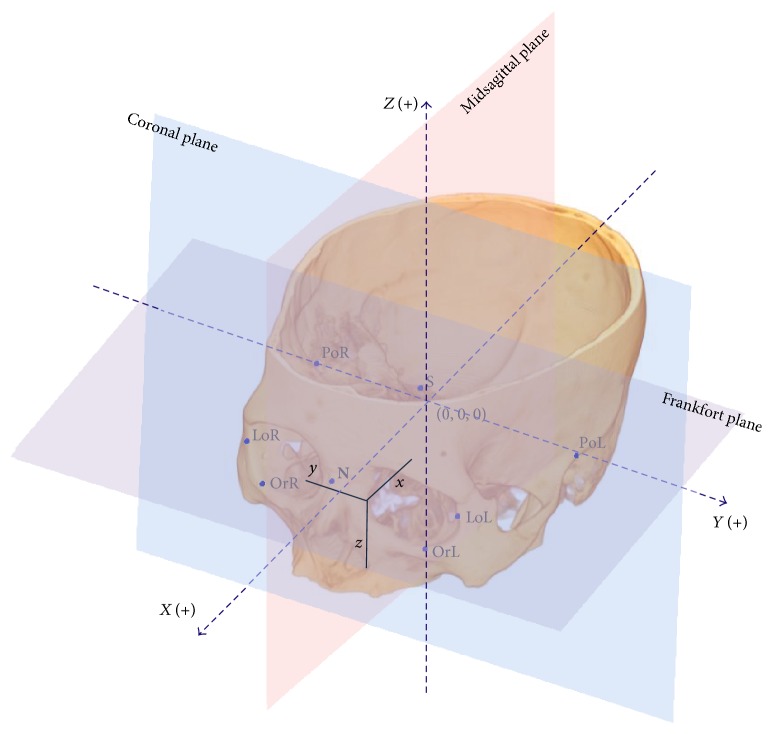
The landmarks and reference coordinate system. PoR = right porion; PoL = left porion; OrR = right orbitale; OrL = left orbitale; N = nasion; S = sella; LoR = right lateral orbital point; LoL = left lateral orbital point. The positive *x*, *y*, and *z* coordinate values indicated the front, left, and superior orientation, respectively.

The volume-rendering image was reconstructed in MIMICS, and then a 3D coordinate system was established based on the landmarks and reference planes described in Table 1. The landmarks were pointed to the volume-rendering image and adjusted on the axial, coronal, and sagittal images. The intersection of the three reference planes was set as the origin (0, 0, 0), and positive *x*, *y*, and *z* coordinate values indicated the front, left, and superior orientations, respectively (Figure 1).

The points of the corneal apex (C) and the eyeball center (E) were marked using the following procedure. The cursor was adjusted and positioned on the middle point of the cornea section in the axial and sagittal images simultaneously; this was then named as point C. A sphere was created with the CAD (computer-aided design) function and was made to overlap with the eyeball wall on the axial, sagittal, and coronal images; then, the center of this sphere was named point E. A 3D vector $\vec{EC}$ was defined to present the position of the globe in the reference coordinate system.

The coordinate of $\vec{EC}$ in the reference coordinate system could be calculated with mathematical methods and expressed as an actual size in millimeters: the STL coordinates of the reference landmarks were recorded, and the equations of the three reference planes could be calculated based on these coordinates as in Table 2. The distance (*d*) from the points C and E to the coronal, midsagittal (MS), and Frankfort horizontal (FH) planes was equal to their coordinates in the reference coordinate system and recorded as (*x*_C_, *y*_C_, and *z*_C_) and (*x*_E_, *y*_E_, and *z*_E_), respectively:

(1) $$\begin{array}{c} d=\frac{\left|Ax+By+Cz+D\right|}{\sqrt{A^{2}+B^{2}+C^{2}}}; \end{array}$$

here, *A*, *B*, *C*, and *D* represent the constant coefficients of the plane equation, and *x*, *y*, and *z* represent the STL coordinates of points E and C.

The angle of $\vec{EC}$ and the FH plane (arg*F*) or the MS plane (arg*M*) was calculated as follows:

(2) $$\begin{array}{c} \frac{\arg F}{\arg M}=\sin^{-1}\left(\frac{\vec{EC}\cdot \vec{n}}{\left|\vec{EC}\right|\left|\vec{n}\right|}\right); \end{array}$$

here, $\vec{n}$ represents the normal vector of the reference plane.

The angle of $\vec{EC}$ and axes $\vec{x}$, $\vec{y}$, and $\vec{z}$ (arg*X*, arg*Y*, and arg*Z*) was calculated as follows:

(3) $$\begin{array}{c} \arg X=\cos^{-1}\left(\frac{\vec{EC}\cdot \vec{x}}{\left|\vec{EC}\right|\left|\vec{x}\right|}\right),\\ \arg Y=\cos^{-1}\left(\frac{\vec{EC}\cdot \vec{y}}{\left|\vec{EC}\right|\left|\vec{y}\right|}\right),\\ \arg Z=\cos^{-1}\left(\frac{\vec{EC}\cdot \vec{z}}{\left|\vec{EC}\right|\left|\vec{z}\right|}\right). \end{array}$$

All calculations were preset using Microsoft Excel software, and the results could be automatically acquired after inputting the original STL coordinate data. All the measurements were made by the same oculoplastic specialist.

### 2.4. Clinical Examination

Hertel exophthalmometry was performed by an experienced ophthalmologist using the standard method. Hirschberg corneal reflex test was performed before CT scanning by the same ophthalmologist; additionally, the objective strabismus angles were measured by synoptophore and recorded.

### 2.5. Statistical Analysis

Statistical analysis was performed using SPSS. Mean values with standard deviations (SD) were given. Twenty patients were randomly selected for interobserver and intraobserver reliability analysis. 2D CT measurement, landmark positioning, and $\vec{EC}$ calculation were repeated by the same observer as well as by another observer without referring to the previous results. The absolute difference between the two measurements was analyzed. Intraclass correlation coefficient (ICC) and Bland-Altman plot were used to evaluate method error. A paired *t*-test was used to assess the paired data, and a nonparametric test was used to compare the other data. Pearson correlation was used to analyze the correlation between data. Linear regression analysis was used to analyze the relativity. *p* < 0.05 was considered statistically significant.

## 3. Results

### 3.1. Measurement Reliability Analysis

For repeated measurements on the same scan, the intraobserver and interobserver agreements are listed in Table 3. Among the patients, 13 underwent unilateral orbital decompression surgery and were given a CT examination pre- and postoperatively within 2 weeks. All 13 of these patients were in the inactive phase, and we assumed that their nonoperated eyes would remain in the same positions; the average absolute difference in $\vec{EC}$ coordinates between the two scans ranged from 0.24 to 0.60 mm, and the average absolute difference was 1.32 mm for 2D exophthalmos measurements. The absolute difference of the 2D exophthalmos between the two scans was significantly larger than $\vec{EC}$ (*p* ranged from 0.001 to 0.008).

### 3.2. $\vec{EC}$ Parameter Analysis

**Figure 2:**
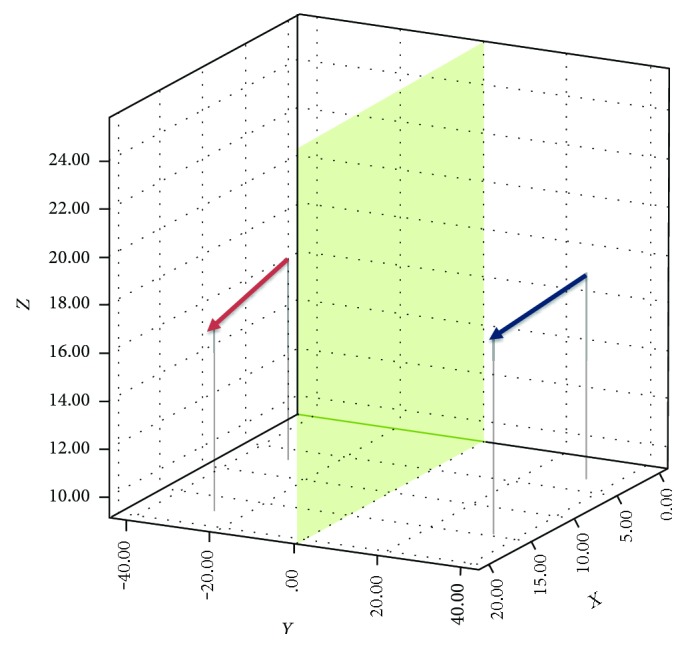
The eyeball vector diagram. The arrowhead represents point C, and the other point of the arrow represents point E. The red and blue arrows represent $\vec{EC}$ of the right and left eye, respectively, and it could be viewed and compared in the 3D coordinate system from different directions.

The vector of the two eyes could be visualized with software and observed and compared from different orientations (Figure 2). For the 61 cases without a postsurgery change, the coordinates and deviation angles of $\vec{EC}$ are listed in Table 4.

### 3.3. $\vec{EC}$ and 2D CT Measurement

The coordinate *x*_C_ of $\vec{EC}$ represented the exophthalmos theoretically. The difference between *x*_C_ and the 2D exophthalmos measurement ranged from −2.5 to 3.2 mm, and there was a significant difference between them (*p* = 0.003). The absolute difference was 1.0 ± 0.73 mm, and there were 29 of 81 (35.8%) cases which had an absolute difference of 1 mm or more.

### 3.4. $\vec{EC}$ and Hertel Exophthalmometry

**Figure 3:**
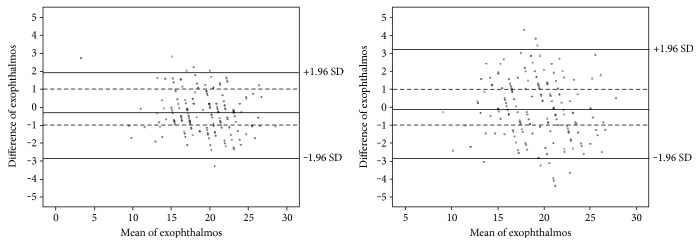
Bland-Altman plots compared the results of Hertel exophthalmometer and CT measurement. The left one showed the difference between the Hertel results and the coordinate *x*_E_ of $\vec{EC}$, and the right one showed the difference between the Hertel results and 2D CT exophthalmos. It could be found that the difference between the Hertel results and 2D CT exophthalmos was more dispersed than that between the Hertel results and *x*_E_ and had much fewer plots within −1~1 mm.

The exophthalmos measured by Hertel exophthalmometer was 19.1 ± 3.6 mm (ranged from 9 to 28 mm). The absolute difference between the Hertel measurement and 2D CT exophthalmos was 1.39 ± 0.97 mm, significantly larger than the difference between the Hertel measurement and *x*_C_ (0.95 ± 0.67 mm, *p* < 0.001), and the correlation between the Hertel measurement and *x*_C_ was slightly stronger (*r* = 0.95) than the correlation between the Hertel measurement and 2D CT exophthalmos (*r* = 0.89). The Bland-Altman plots showed that the difference between the Hertel measurement and 2D CT exophthalmos was more dispersed than that between the Hertel measurement and *x*_C_ and had much fewer plots within −1~1 mm (Figure 3).

### 3.5. $\vec{EC}$ and Clinical Strabismus Angle

Hirschberg test was performed by an experienced ophthalmologist, and the results ranged from 0 to 45° (15.3° ± 12.4°). The direction of strabismus was compared with the vector orientation, and no significant difference was found between them (*p* = 0.33). The values of arg*X* were also compared with the angles of Hirschberg test, and there was no significant difference between them (*p* = 0.52); the correlation between them was strong (*r* = 0.87).

The synoptophore test was performed in 37 cases to record the objective deviation angles: the horizontal deviation degrees ranged from −10 to 40° (10.1° ± 13.2°), with a positive value representing esotropia and a negative value representing exotropia; the vertical deviation ranged from 0 to 35° (9.2° ± 10.2°), with a positive value representing hypertropia and a negative value representing hypotropia. The values of arg*F* were compared with the vertical deviation angles tested by synoptophore, and there was no significant difference between them (*p* = 0.16); the correlation between them was strong (*r* = 0.86). The correlation between the values of arg*M* and the horizontal deviation angles was strong (*r* = 0.98), but the angles tested by synoptophore were significantly larger than those tested by arg*M* (including both positive and negative values, *p* = 0.001).

### 3.6. $\vec{EC}$ and Eyeball Movement

The arg*M* values between the two eyes were compared, and it was found that in the eyes with a larger arg*M* (including positive and negative values), the absolute values of *y*_C_ were significantly smaller than the values of their fellows (*p* < 0.001); in the eyes with a smaller arg*M*, the absolute values of *y*_C_ were significantly larger than those of their fellows (*p* < 0.001), and there was a strong negative correlation between the difference of arg*M* and the difference of absolute values of *y*_C_ (*r* = −0.94, *p* < 0.001). The values of arg*F* were also compared: in the eyes with a larger arg*F*, the values of *z*_C_ were significantly larger than those of their fellows (*p* = 0.007); in the eyes with a smaller arg*F*, the values of *z*_C_ were significantly smaller than those of their fellows (*p* < 0.001), and there was a strong correlation between the difference of arg*F* and the difference of *z*_C_ (*r* = 0.808, *p* < 0.001). However, no significant difference was found in the absolute values of *y*_E_ or the values of *z*_E_ between the two eyes (*p* ranged from 0.08 to 0.96).

**Figure 4:**
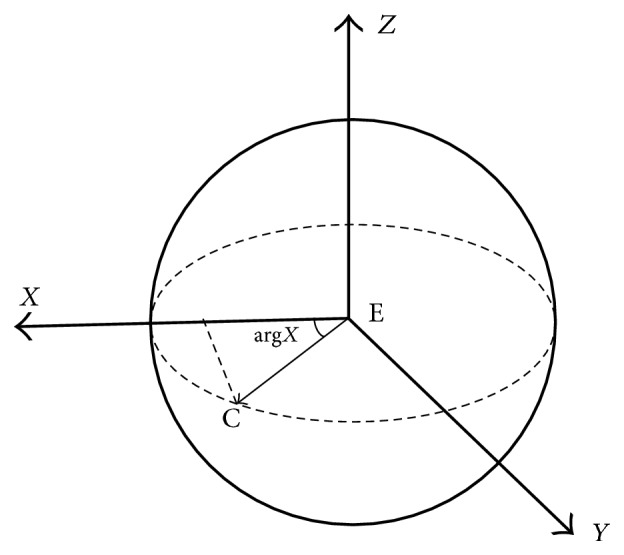
The geometrical relationship between the corneal apex and the eyeball center. Theoretically, $x_{C}=\cos\left(\arg X\right)\times \left|\vec{EC}\right|+x_{E}$. Similarly, the calculation equation could be acquired for *y*_C_ and *z*_C_.

**Figure 5:**
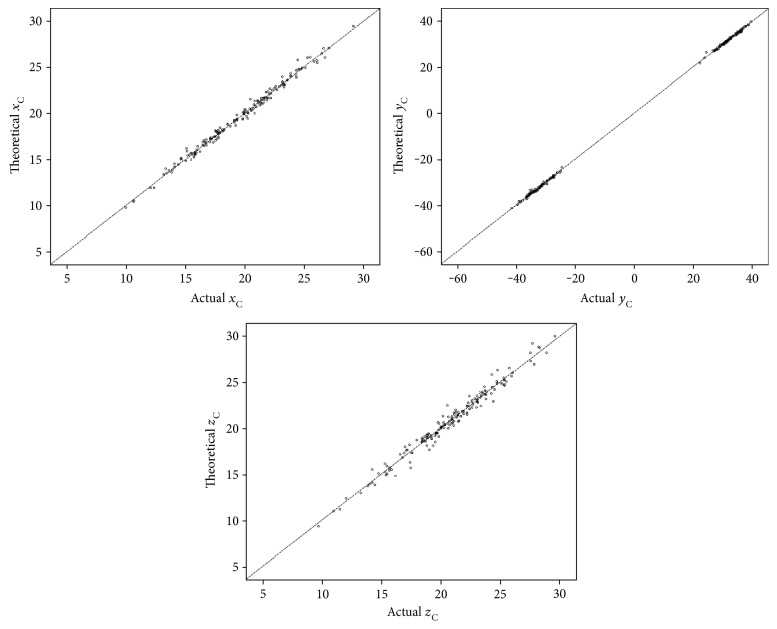
The scatterplots about the theoretical and actual coordinates of the corneal apex. The dotted line represents the equation *y* = *x*, and we could find that the theoretical values were consistent with the actual values near perfect.

In theory, the relation between points E and C should be as follows: $x_{C}=\cos\left(\arg X\right)\times \left|\vec{EC}\right|+x_{E}$; $\;y_{C}=\cos\left(\arg Y\right)\times \left|\vec{EC}\right|+y_{E}$; and $z_{C}=\cos\left(\arg Z\right)\times \left|\vec{EC}\right|+z_{E}$ (Figure 4). We calculated the theoretical values of *x*_C_, *y*_C_, and z_C_ based on these equations, and there was no significant difference between the theoretical *x*_C_ and *x*_C_ (*p* = 0.46, *r* = 0.995), the theoretical *y*_C_ and *y*_C_ (*p* = 0.21, *r* = 1.0), or the theoretical *z*_C_ and *z*_C_ (*p* = 0.29, *r* = 0.988). The linear regression equations of the theoretical *x*_C_, *y*_C_, and *z*_C_ and their actual values were all *y* = *x* (*b* = 1 with no constant, *p* < 0.001) (Figure 5).

## 4. Discussion

In previous research on TED, there have been few references to the change in the axial globe position in vertical and lateral orientations, and measurement methods have not been uniform: horizontal eye positions are sometimes evaluated using the interpupillary distance [7] and sometimes using the distance between the most medial part of the eyeballs measured on an axial CT image [6]. Fichter et al. examined the horizontal position of the eyeball by measuring the distance between the nasion and the pupil using a digital pupillometer [8]. Alsuhaibani et al. measured the vertical difference between the two eyeballs on coronal CT images to evaluate the vertical position change [6]. However, nearly all of these methods only evaluated the relative position between the two eyes.

Based on 3D reconstructed images, we tried to describe the axial globe position in more directions. The establishment of a craniofacial 3D coordinate system has been described in many reports [11–14]; in this study, we chose the lateral orbital rim to set the coronal plane because it could make the coordinates consistent with the clinical exophthalmos measurement. The reproducibility of the reference coordinate system was proven stable, which is consistent with previous studies [11, 15]. The coordinates of $\vec{EC}$ were obtained by preset mathematical calculation rather than measuring images, which made the process more accurate and convenient. Besides the same CT scan, we assessed the measurement reliability of different CT scans, which has not been discussed before. Our results indicated that the reproducibility of $\vec{EC}$ was significantly better than the 2D CT measurement. Additionally, the coordinate *x*_E_, which represented the exophthalmos, was significantly different from exophthalmos values obtained from the 2D CT images, and in more than one third of cases differed by more than 1 mm, which was considered an accepted error between the repeated exophthalmometry by different observers [16]. The difference between Hertel exophthalmos and 2D CT measurement was found to be variable in previous reports [3, 17, 18]. In our study, the consistency between $\vec{EC}$ and Hertel exophthalmos was better than consistency between 2D CT measurement and Hertel exophthalmos. This might be because the stability of the 3D coordinate system reduces head position change and measurement error, especially when following up patients using different CT scans.

Vector analysis has been used in some research [11, 19, 20], but this study represents the first time that a vector has been used to define the position of the eyeball in TED. A 3D vector can give a more comprehensive and intuitive view of the globe position. In previous reports, the mean values of 2D CT exophthalmos ranged from 21.2 mm to 22.5 mm in TED [1, 3, 21]; in our study, the mean value was 19.5 mm. The mean distance between the nasion and the pupillary center was 33.8 mm in TED [8], and the mean distance between the corneal apex and the MS plane was 32.2 in our study. The mean vertical height of the corneal apex from the FH plane was 20.8 mm in our study. Additionally, we have also listed the location of the eyeball center in Table 4. These results might give a new reference for the axial globe position research in TED.

The vector $\vec{EC}$ can describe the deviation of the eyeball. We assumed that $\vec{EC}$ approximated the ocular axis and that its normal position was approximately parallel to the *x*-axis; the angles of $\vec{EC}$ then indicated the severity of eyeball deviation in different directions. The orientation and angle of $\vec{EC}$ were largely in accordance with Hirschberg test; however, depending on the experience of the ophthalmologist, the Hirschberg test gave a rough value while $\vec{EC}$ could give a more exact deviation angle. The vertical deviation of $\vec{EC}$ was also in accordance with the synoptophore results. However, the horizontal deviation tested by synoptophore was significantly larger than that of the $\vec{EC}$ in the esotropia patients and smaller in the exotropia patients, although there was a very strong correlation between them. This difference might be because the synoptophore was affected by the artificial viewing conditions, resulting in overconvergence [22]. Although there were some other factors that might have affected the clinical strabismus examination and $\vec{EC}$ measurement, such as the angle kappa, convergence, test distance, and measurement error, our study still indicated that, to some extent, the deviation of the eyeball as measured by morphological method was consistent with that found in clinical examination and might better reflect the anatomical position of the eyeball.

When an eye had a larger arg*M* than its fellow, meaning that the eyeball had a more severe medial tilt; its corneal apex was closer to the nasal side than its fellow. When an eye had a larger arg*F*, meaning that the eyeball had a more severe superior tilt, its corneal apex was higher than its fellow. However, there was no significant change in the positions of the eyeball center. The results indicated that the tilt of eyeball mainly depended on a rotation motion around its center. The fibrotic extraocular muscles pulled the eyeball [23, 24] and made it rotate around the eyeball center, making the corneal apex change its position in addition to protruding, but the lateral or vertical shift of the eyeball center was not obvious. Of course, the influence came from extrusion of the expanded orbital tissue, and the shift during ocular ductions could not be excluded completely [25]. Additionally, due to changes in orbital bone, fat, and extraocular muscles, eyeball motion can be complicated during TED surgery, possibly leading to changes in strabismus or exophthalmos [26, 27]. The vector method might help us to better study the effects of surgery better in TED patients, including eyeball shift and rotation.

To further discuss the problem mentioned above, the relativity between the corneal apex and the eyeball center was analyzed. The relative position of the corneal apex to the eyeball center was influenced by the deviation angles of $\vec{EC}$, suggesting that the bilateral corneal apexes might be asymmetrical even if the eyeball centers were symmetrical. The deviation of the corneal apex could disturb the exophthalmos measurement both using Hertel exophthalmometer and 2D CT images; the vector would improve this problem.

Although there were some drawbacks to the vector method, for example, it could not reflect the globe rotation around the ocular axis, it still supplied a novel and useful way of measuring the axial globe position.

In conclusion, the establishment of a 3D coordinate system and globe vector is a feasible and reliable method of depicting the axial globe position in TED. The vector method can depict the globe position in a more intuitive and comprehensive way and can provide more information about the eyeball location and its deviation than traditional methods. Additionally, vector analysis might help evaluate eyeball motion more accurately after decompression surgery in TED.

## Figures and Tables

**Table 1:** The landmarks, reference points, and planes of the 3D coordinate system.

		Abbreviation	Definition
Landmark	Right porion	PoR	Highest midpoint on roof of the right skeletal external auditory meatus
	Left porion	PoL	Highest midpoint on roof of the left skeletal external auditory meatus
	Right orbitale	OrR	Lowest point on the right infraorbital margin
	Left orbitale	OrL	Lowest point on the left infraorbital margin
	Nasion	N	The point of contact between the frontal bone and suture between 2 halves of the nasal bones
	Sella	S	The midpoint of the pituitary fossa of the sphenoid bone
	Right lateral orbital point	LoR	The deepest point on the right lateral orbital rim
	Left lateral orbital point	LoL	The deepest point on the left lateral orbital rim

Reference point	Middle point of porion	PoM	The middle point of the right and left porion
	Middle point of orbitale	OrM	The middle point of the right and left orbitale
	Middle point of lateral orbital point	LoM	The middle point of lateral orbital point

Reference plane	Frankfort horizontal plane (*xy* plane)	FH plane	Passing through PoR, PoL, and OrM
	Midsagittal plane (*xz* plane)	MS plane	Passing through N and S and being perpendicular to the FH plane
	Coronal plane (*yz* plane)		Passing through LoM and orthogonal to the MS and FH plane

**Table 2:** The calculation equations of the reference planes. All the coordinate values used for calculation were STL coordinates of MIMICS.

Reference plane	Plane equation	Calculated equation of coefficients
FH plane	*A*_F_ *x* + *B*_F_*y* + *C*_F_*z* + *D*_F_ = 0	*A*_F_ = (*y*_PoL_ − *y*_PoR_)(*z*_OrM_ − *z*_PoR_) − (*y*_OrM_ − *y*_PoR_)(*z*_PoL_ − *z*_PoR_)
		*B*_F_ = (*z*_PoL_ − *z*_PoR_)(*x*_OrM_ − *x*_PoR_) − (*z*_OrM_ − *z*_PoR_)(*x*_PoL_ − *x*_PoR_)
		*C*_F_ = (*x*_PoL_ − *x*_PoR_)(*y*_OrM_ − *y*_PoR_) − (*x*_OrM_ − *x*_PoR_)(*y*_PoL_ − *y*_PoR_)
		*D*_F_ = −A_F_ × *x*_PoR_ − *B*_F_ × *y*_PoR_ − *C*_F_ × *z*_PoR_

MS plane	*A*_M_ *x* + *B*_M_*y* + *C*_M_*z* + *D*_M_ = 0	*A*_M_ = (*y*_S_ − *y*_N_) × *C*_F_ − (*z*_S_ − *z*_N_) × *B*_F_
		*B*_M_ = (*z*_S_ − *z*_N_) × *A*_F_ − (*x*_S_ − *x*_N_) × *C*_F_
		*C*_M_ = (*x*_S_ − *x*_N_) × *B*_F_ − (*y*_S_ − *y*_N_) × *A*_F_
		*D*_M_ = −*A*_M_ × *x*_N_ − *B*_M_ × *y*_N_ − *C*_M_ × *z*_N_

Coronal plane	*A*_C_ *x* + *B*_C_*y* + *C*_C_*z* + *D*_C_ = 0	*A*_C_ = *B*_F_ × *C*_M_ − *C*_F_ × *B*_M_
		*B*_C_ = *C*_F_ × *A*_M_ − *A*_F_ × *C*_M_
		*C*_C_ = *A*_F_ × *B*_M_ − *B*_F_ × *A*_M_
		*D*_C_ = −*A*_C_ × *x*_LoM_ − *B*_C_ × *y*_LoM_ − *C*_C_ × *z*_LoM_

**Table 3:** The reliability analysis of CT measurement.

	Intraobserver						Interobserver					
	Mean of absolute difference (mm)			ICC			Mean of absolute difference (mm)			ICC		
	*x*-axis	*y*-axis	*z*-axis	*x*-axis	*y*-axis	*z*-axis	*x*-axis	*y*-axis	*z*-axis	*x*-axis	*y*-axis	*z*-axis
Landmark												
N	0.76	0.46	0.4	0.996	0.999	0.998	0.98	0.54	0.41	0.993	0.993	0.989
S	0.89	0.91	0.43	0.998	0.999	0.997	0.56	0.96	0.89	0.994	0.995	0.991
PoR	0.5	0.7	0.87	0.998	0.997	0.995	0.63	0.82	1.01	0.987	0.984	0.991
PoL	0.5	0.71	0.96	0.999	0.998	0.993	0.69	0.93	0.98	0.991	0.984	0.993
OrR	0.52	0.41	0.97	0.999	0.999	0.994	0.42	0.37	1.02	0.989	0.986	0.968
OrL	0.57	0.49	0.92	0.999	0.999	0.994	0.45	0.41	0.99	0.991	0.993	0.972
LoR	0.71	0.33	0.57	0.998	0.996	0.997	0.68	0.39	0.52	0.981	0.987	0.983
LoL	0.64	0.33	0.48	0.997	0.999	0.996	0.59	0.46	0.54	0.992	0.989	0.978
Corneal apex	0.58	0.58	0.51	0.993	0.988	0.975	0.53	0.47	0.57	0.983	0.981	0.972
Eyeball center	0.59	0.48	0.69	0.988	0.979	0.964	0.56	0.62	0.71	0.981	0.976	0.978
2D exophthalmos	0.48			0.993			0.54			0.987		

**Table 4:** The coordinates and angles of the vector $\vec{EC}$. The *y*_C_ and *y*_E_ of the left eyes were negative, and their absolute values were used. The positive values of arg*M* represent the ocular axis turned medial, and the negative represent turned outward. The positive values of arg*F* represent the ocular axis turned up, and the negative values represent the opposite.

	Both eyes			Right eye			Left eye		
	Mean	SD	Range	Mean	SD	Range	Mean	SD	Range
*x*_E_ (mm)	19.5	3.7	10.0~29.1	19.3	4	10.0~27.0	19.7	3.8	12.1~29.1
Absolute *y*_E_ (mm)	32.2	3.7	22.4~41.3	−32.3	3.7	24.3~41.2	32.3	3.6	22.4~39.5
*z*_E_ (mm)	20.8	3.6	10.0~29.6	20.3	3.4	11.2~28.2	21.3	3.8	10.0~29.6
*x*_C_ (mm)	7.6	3.3	0.9~17	7.4	3.1	0.9~14.3	7.8	3.6	1.4~17.0
Absolut *y*_C_ (mm)	32.3	2.4	27.7~38	−32.1	2.4	27.8~37.0	32.5	2.4	27.7~38.0
*z*_C_ (mm)	20	1.8	16.0~24.3	19.9	1.7	16.5~24.3	20.1	1.9	16.0~24.3
arg*M* (°)	0.6	11.8	−45.5~35.4	−0.6	12.9	−45.5~27.5	1.1	10.1	−16.5~35.4
arg*F* (°)	4	13.7	−48.8~41.2	2.3	13.8	−37.8~41.2	5.7	13.6	−48.8~39.2
arg*X* (°)	15.3	8.4	1.6~49.1	15.4	8.8	1.7~41.8	15.2	8.3	1.6~49.1
